# Dynamic Adoption of Immersive Tai Chi Training Among Older Adults: Exploratory Qualitative-Dominant Longitudinal Mixed Methods Follow-Up Study of Virtual and Mixed Reality Delivery

**DOI:** 10.2196/96262

**Published:** 2026-09-15

**Authors:** Xiacheng Song, Huafeng Qu, Jing Jin, Lu Sun, Xiqiong Yi, Junfeng Zhu, Huirong Huang

**Affiliations:** 1 Guangdong Industry Polytechnic University, Guangzhou, Guangdong, China; 2 Information and Intelligent Engineering School, Yunnan College of Business Management, Vocational Education Park, Anning City, No. 17 Qilin Road, Kunming, Yunnan, 650300, China, 86 13398718004; 3 Guangdong University of Science and Technology, Dongguan, Guangdong, China; 4 Xi'an Fanyi University, Xi'an, Shaanxi, China; 5 Dongguan Polytechnic, Dongguan, Guangdong, China; 6 Zhaoqing University, Zhaoqing, Guangdong, China

**Keywords:** older adults, virtual reality, mixed reality, Tai Chi, exergame, technology adoption, mixed methods, qualitative research

## Abstract

**Background:**

Immersive exercise systems may support guided movement practice and physical activity among older adults, but adoption of head-mounted systems cannot be inferred from single-session usability or intention ratings. Older adults must negotiate unfamiliar equipment, movement safety, comfort, perceived control, trust, and perceived benefit. Virtual reality (VR) and mixed reality (MR) are often grouped as extended reality despite different sensory and safety affordances.

**Objective:**

This study examined how older adults’ barriers and facilitators evolved during repeated immersive Tai Chi training and how VR and MR delivery appeared to shape immersion, control, trust, safety appraisal, and conditional willingness to continue use.

**Methods:**

We conducted an exploratory, qualitative-dominant longitudinal mixed methods follow-up study in Hunan, China. Community-dwelling adults aged 60 years or older completed guided Tai Chi training in VR or MR while retaining parent-study allocation; comparisons were descriptive and hypothesis-generating. The completed-protocol quantitative sample and the main qualitative denominator each included 34 participants (VR: n=16; MR: n=18). Data sources comprised semistructured interviews, Game Experience Questionnaire dimensions, Virtual Reality Sickness Questionnaire (VRSQ) scores, safety observations, and task performance logs from days 1, 3, and 6. Qualitative data were coded using a Dynamic Barrier Framework–informed codebook and integrated with descriptive quantitative summaries.

**Results:**

Adoption unfolded as a staged process. During entry, unfamiliarity, competence concerns, and mild physical discomfort shaped responses. Initial uncertainty was endorsed by 7 of 16 (43.8%, 95% CI 23.1‐66.8) VR participants and 6 of 18 (33.3%, 95% CI 16.3‐56.3) MR participants. During adaptation, participants described practice, feedback, social reinforcement, and environmental experience; accuracy improved from day 1 to day 6 by 0.083 (95% CI 0.024‐0.142) in VR and 0.103 (95% CI 0.037‐0.169) in MR. Late-stage willingness remained conditional on comfort, safety, content variety, and usefulness. VR accounts more often emphasized environmental immersion and affective value, whereas MR accounts more often emphasized reality grounding, perceived control, and trust. Standardized VRSQ scores were low overall (VR 8.65 and MR 5.65, on a 0‐100 scale), with no falls, serious adverse events, or session terminations.

**Conclusions:**

This study combines repeated qualitative accounts with symptom, safety, and performance evidence while holding the Tai Chi content constant across VR and MR delivery. Unlike single-session acceptance studies, broad extended reality comparisons, or endpoint-only evaluations, it shows adoption as a phase-sensitive and conditional process rather than a one-time decision. The Dynamic Barrier Framework explains how entry barriers may soften, persist, or change meaning and how modality-sensitive affordances shape experience without implying device superiority. In practice, immersive exercise should combine gradual orientation, monitored exposure, comfort and safety safeguards, understandable feedback, social support, and modality-sensitive design. Longer community or home follow-up remains necessary before inferring sustained adherence or unsupervised safety.

## Introduction

### Problem and Description of Research Question

Population aging has increased the need for scalable approaches that support balance, mobility, confidence, and continued physical activity among older adults [1,2]. Falls and fall-related functional decline remain major public health concerns, and exercise-based prevention strategies are consistently recommended for community-dwelling older adults [3,4]. Tai Chi is especially relevant in this context because it combines weight shifting, coordinated postural transitions, attentional focus, and gradual movement control, and evidence continues to support its value for balance-related outcomes in older adults [5,6]. However, conventional Tai Chi training depends on access to instruction, safe practice environments, and repeated attendance. These requirements can be difficult for older adults who face mobility limitations, limited local resources, variable confidence, or practical barriers to sustained participation [7,8].

### Review of Relevant Scholarship

Immersive exercise systems have been proposed as one way to extend guided practice beyond conventional rehabilitation and group-class settings. Head-mounted systems can provide structured guidance, immediate feedback, and repeatable practice, and recent syntheses report promising effects on balance and physical activity in older adults [9,10]. However, those reviews also identify heterogeneous interventions, short follow-up, small samples, and unresolved usability issues [9,10]. Evidence that an immersive program can be completed or can improve a physical endpoint therefore does not by itself explain whether older adults will continue to use it, why their judgments change, or what implementation conditions make continued use acceptable. Earlier clinical, home-use, acceptance, virtual reality (VR), and augmented reality (AR) feasibility studies similarly show that readiness depends on usability, context, and age-appropriate implementation [11-16]. Broader medical-VR reviews also show substantial variation in applications and use contexts, reinforcing the need for context-specific adoption evidence [17].

The first gap is temporal. Technology acceptance models, including the technology acceptance model (TAM), the unified theory of acceptance and use of technology (UTAUT), diffusion of innovations, and continuance models, explain usefulness, ease of use, social influence, and intention [18-23], but immersive exercise acceptance is still commonly assessed at a single time point or as a pre-post score. Recent studies show that Chinese older adults can report usefulness and enjoyment after immersive fall-prevention training and that acceptance can remain high after repeated group VR exposure [24,25]. What remains underexplored is how an initial lack of familiarity becomes confidence, trust, conditional willingness, or continued concern during repeated movement practice. This distinction matters because first-session hesitation may be an appropriate appraisal of balance, vision, headset comfort, and task control rather than stable rejection of the technology.

The second gap concerns modality and mechanism. VR typically occludes the physical environment, whereas mixed reality (MR) preserves visual access to the surrounding room through passthrough while overlaying digital training elements [26-28]. A recent preliminary trial suggests that MR exercise can be feasible and beneficial for older adults with sarcopenia [29], but it compared MR with conventional exercise rather than examining VR and MR versions of the same activity. Broad extended reality (XR) categories and endpoint comparisons cannot show how environmental occlusion, visual grounding, immersion, perceived control, and trust are experienced over time. Direct, repeated-exposure evidence on these modality-sensitive processes remains limited.

The third gap is social and contextual. Repeated group VR and remote group–mediated physical activity studies indicate that caregivers, trusted individuals, peers, and group delivery can influence participation and acceptance [25,30]. A recent six-session qualitative study likewise found that older adults could enjoy a VR exercise game while still requesting greater challenge, physical demand, and social engagement [31]. Adoption is therefore coproduced by the device, the exercise task, feedback, supervision, and the community setting; it cannot be reduced to visual immersion or individual intention alone.

A related implementation problem is that short-term attendance is often treated as adherence. A 2026 review found that XR exercise studies for older adults rarely tracked long-term engagement and inconsistently measured exercise intensity and accuracy, with few studies conducted in community settings [32]. This creates a mismatch between claims that a system is acceptable and evidence that it can support sustained, safe, meaningful use. For older adults, the relevant barriers span physical symptoms and fall concerns, technical operation and feedback, psychological control and competence, and social encouragement and support [9,10,32].

We therefore used a Dynamic Barrier Framework (DBF) to organize barriers and facilitators across entry, adaptation, and conditional adoption phases. The DBF follows how a barrier can soften, persist, or change meaning after orientation and repeated supervised exposure. It does not assume a causal pathway and was not used to prove modality superiority. Instead, it structures evidence about unfamiliar equipment, bodily sensations, practice feedback, social reinforcement, perceived control, trust, and future-use conditions. The phase structure was also informed by established distinctions between adoption, maintenance, and stage-based behavior change [33,34].

Accordingly, this study addresses 3 linked gaps: static measurement of a changing acceptance process; limited direct evidence on how VR and MR delivery shape experience when the exercise content is held constant; and insufficient integration of bodily, technical, social, and performance evidence across repeated sessions. Its contribution is a phase-sensitive, qualitative-dominant explanation of adoption conditions, not a new parametric uncertainty model or a confirmatory comparison of device efficacy.

### Study Objectives

No confirmatory hypotheses were specified. The objective of this study was to examine how older adults’ barriers and facilitators evolved during repeated immersive Tai Chi training and how VR and MR delivery appeared to shape immersion, control, trust, safety appraisal, and conditional willingness to continue. Specifically, we asked: (1) What entry-stage concerns and facilitators were described during early exposure? (2) How did practice, feedback, social reinforcement, and environmental experience support adaptation? (3) Under what conditions did participants describe willingness to continue use? The study was designed as an exploratory, qualitative-dominant, longitudinal mixed methods follow-up rather than a confirmatory test of VR versus MR superiority.

## Methods

### Research Design Overview

This was an exploratory, qualitative-dominant longitudinal mixed methods follow-up study of older adults’ experiences with VR- and MR-delivered versions of the same immersive Tai Chi exergame. No single philosophical paradigm was prospectively specified. The analysis was pragmatic and interpretive: participant accounts were treated as situated descriptions of experience, while questionnaires, safety observations, and performance logs were used only for descriptive triangulation. In the parent repeated-exposure experiment, participants were allocated 1:1 to VR or MR using a computer-generated sequence. The current follow-up retained that allocation without rerandomization. Because follow-up participation depended on consent, availability, and protocol completion, all VR and MR comparisons are descriptive and hypothesis-generating rather than confirmatory tests of modality superiority [35].

### Study Participants or Data Sources

#### Researcher Description

Qualitative coding was conducted by the first author (XS) and one additional researcher (HQ). Both researchers worked from the same phase-based codebook and excerpt-level matrix, and the first author checked the Chinese-to-English translations used for reporting. No independent third adjudicator was used; disagreements were resolved through discussion and consensus. Reflexive management included coding memos, comparison of coding decisions, codebook revisions, and retention of original Chinese excerpts alongside translations and interpretations.

#### Researcher-Participant Relationship

Some participants were previously known to members of the research team through community connections, whereas others had no prior relationship with the researchers. Prior familiarity was not used to determine eligibility, group allocation, or coding. Because familiarity could influence willingness to participate or what participants disclosed, the team used the same interview guide, study-code system, phase-based codebook, and consensus procedures across participants and did not treat familiarity as evidence for a preferred interpretation. Sample handling is summarized in Table 1.

**Table 1.** Sample handling for the exploratory qualitative-dominant mixed methods follow-up study.

Item	Value
All interview transcripts identified	35 interview transcripts: completed-protocol VR^a^ (n=16) and MR^b^ (n=18), plus MR37 partial-exposure transcript
Completed-protocol quantitative sample.	34 participants: VR, n=16; MR, n=18
Main completed-protocol qualitative coded evidence	34 participants: VR, n=16; MR, n=18
Partial-exposure qualitative case excluded from main counts	Participant 37 (MR)
Completed-protocol VR interview added to coding matrix	Participant 6 (VR), six author-confirmed paraphrased coding rows; not used as verbatim quotations

a VR: virtual reality.

b MR: mixed reality.

#### Participants or Other Data Sources and Participant Characteristics

The completed-protocol analysis used interview evidence, questionnaire responses, safety observations, and session-level performance exports from 34 community-dwelling older adults (VR, n=16; MR, n=18). One additional MR partial-exposure transcript was retained only as a diagnostic case. Participant characteristics relevant to interpretation, including age, sex, balance, exercise, and technology-use indicators, are reported in Table 2.

**Table 2.** Participant characteristics by group in the complete quantitative sample. This descriptive table is used to interpret baseline imbalance and does not report hypothesis tests.

Variable and level^a^		Overall (N=34)	VR^b^ (n=16)	MR^c^ (n=18)
Age (in years), mean (SD)		68.65 (7.89)	69.38 (8.80)	68.00 (7.17)
Sex, n (%)				
Male		10 (29.4)	7 (43.8)	3 (16.7)
Female		24 (70.6)	9 (56.2)	15 (83.3)
BMI (kg/m²), mean (SD)		23.35 (3.01)	22.92 (2.82)	23.73 (3.19)
Education, n (%)				
Primary school or below		4 (11.8)	2 (12.5)	2 (11.1)
Middle school		13 (38.2)	4 (25.0)	9 (50.0)
High school or technical secondary		13 (38.2)	7 (43.8)	6 (33.3)
Junior college or above		4 (11.8)	3 (18.8)	1 (5.6)
Daily smartphone use, n (%)				
<1 hour		4 (11.8)	2 (12.5)	2 (11.1)
1‐3 hours		9 (26.5)	3 (18.8)	6 (33.3)
3‐5 hours		12 (35.3)	9 (56.2)	3 (16.7)
≥5 hours		9 (26.5)	2 (12.5)	7 (38.9)
BBS^d^, mean (SD)		54.47 (3.83)	53.81 (5.06)	55.06 (2.26)
Exercise intensity, n (%)				
Low		6 (17.6)	5 (31.2)	1 (5.6)
Light		9 (26.5)	3 (18.8)	6 (33.3)
Vigorous		19 (55.9)	8 (50.0)	11 (61.1)
Health status, n (%)				
No diagnosed disease		14 (41.2)	6 (37.5)	8 (44.4)
Cardiopulmonary disease		20 (58.8)	10 (62.5)	10 (55.6)

a Values are mean (SD) for continuous variables and n (%) for categorical variables. Percentages are calculated within each column.

b VR: virtual reality.

c MR: mixed reality.

d BBS: Berg Balance Scale.

#### Inclusion and Exclusion and Participant Selection

Participants were community-dwelling older adults recruited through local community centers and community-based outreach in Hunan, China. Eligibility criteria included age 60 years or older, ability to stand independently, ability to follow guided Tai Chi movements, and willingness to use a head-mounted display under supervision. Exclusion criteria included conditions that could increase risk or compromise safe participation, such as severe vertigo or motion-sickness susceptibility, history of seizure, severe uncorrected visual or auditory impairment, uncontrolled cardiovascular disease, severe musculoskeletal limitations that prevented safe standing movement practice, inability to provide informed consent, or other contraindications identified during screening.

### Participant Recruitment

#### Sampling Procedures and Recruitment Process

The broader research program initially contacted 86 community-dwelling older adults through community-center promotion, on-site outreach, local newspaper advertising, and community contact networks in Hunan, China. After screening, 70 participants completed the initial single-session experiment; 40 of these participants voluntarily entered the repeated-exposure follow-up from which the current interview and session evidence was drawn. The archived follow-up materials contained 35 interview transcripts. For the current analysis, the completed-protocol quantitative and session sample and main qualitative coded sample each included 34 participants (VR, n=16; MR, n=18). Participant 37 was retained only as an MR partial-exposure diagnostic case and excluded from main theme counts. Participant 6 was a completed-protocol VR participant whose author-confirmed paraphrased coding contributed to participant-level theme counts but was not eligible for use as a verbatim quotation.

#### Sample Size, Power, and Precision

The sample size was determined by feasibility, participant availability, and the qualitative-dominant purpose of the study. The study was not powered to test VR and MR superiority or causal mechanisms. Quantitative estimates are presented with 95% CIs to describe precision and to support triangulation with qualitative findings. Small denominators, baseline imbalance, and retained allocation are treated as constraints on interpretation rather than as defects that can be overcome through statistical modeling.

#### Conditions and Design and Training Conditions and Procedure

Both conditions used the same Tai Chi exergame content delivered through a Meta Quest 3 head-mounted display. The VR condition presented a fully virtual environment that occluded the surrounding physical environment. The MR condition used full-color video passthrough with virtual overlays, allowing participants to retain visual access to the real environment. The same application build, task goals, and core settings were used across conditions; delivery mode was the intended difference.

The exergame implemented a simplified 16-form Tai Chi sequence guided by a virtual coach. For each movement, participants aligned the head-mounted display and the left and right controllers with 3 predefined target hotspots. Holding any hotspot within the success window for at least 3 seconds counted as one successful hotspot hold, and 48 successful holds represented one complete 16-movement loop. The sequence restarted when a loop was completed before the session ended. Participants received immediate visual guidance and end-of-session performance summaries. The repeated-exposure protocol comprised 6 sessions delivered across 6 consecutive days, with one session per day; days 1, 3, and 6 were the standardized performance checkpoints used in this analysis.

#### Masking

Masking was not applicable. Participants and research staff could see whether VR or MR was delivered. The follow-up retained the parent-study allocation, and all comparisons were descriptive.

### Measures and Covariates

#### Instrumentation

Baseline variables included age, sex, education, daily smartphone use duration, Berg Balance Scale (BBS) [36], self-reported exercise intensity, self-reported health status, height, weight, and body mass index. Subjective experience was summarized using a Chinese-adapted 32-item version of the Game Experience Questionnaire (GEQ) core module [37]. Responses were coded on the 0‐4 analytic scale. The standard story-interest item was not administered; therefore, sensory and imaginative immersion was calculated from 5 items rather than the standard 6. The remaining analyzed dimensions were competence, flow, tension and annoyance, challenge, negative affect, and positive affect. Cybersickness was assessed with the 9-item Virtual Reality Sickness Questionnaire (VRSQ) [38]. The oculomotor score was calculated as the sum of items 1, 2, 4, and 5 divided by 12 and multiplied by 100; the disorientation score was calculated as the sum of items 3 and 6‐9 divided by 15 and multiplied by 100. The standardized VRSQ total was the mean of these two subscale scores. Automated game logs provided average accuracy rate and average time spent at standardized checkpoints on days 1, 3, and 6. Average accuracy rate was the proportion of logged frames in which the head and hand hotspots were within the game’s tolerance window. Average time spent was recorded in seconds, with lower values indicating faster task performance [39]. Trajectory was retained only as a diagnostic field because its direction and operational definition could not be verified and was therefore excluded from manuscript-ready analyses.

#### Quality of Measurements

Measurement quality was evaluated using item-level completeness, observed score ranges, floor and ceiling concentrations, and consistency between questionnaire scoring rules and the available item data. Performance-field definitions and units were checked against the available game documentation and prior study records.

#### Psychometrics

Within-sample Cronbach alpha was reported for analyzed GEQ dimensions and the VRSQ where appropriate. Test-retest reliability was not estimated because repeated administrations represented changing session experiences rather than a stability assessment.

### Data Collection

#### Data Collection Procedures

Semistructured interviews were conducted by the research team across the repeated-exposure follow-up using selected postsession interview modules rather than a single endpoint interview. Interviews were audio recorded and generally lasted approximately 20 minutes. The interview guide prompted participants to describe initial impressions, unfamiliarity, bodily sensations, task difficulty, environmental experience, perceived safety, social reinforcement, perceived learning, trust, and willingness to continue use. Modules were aligned with early contact, adaptation after repeated practice, and later-session evaluation, so later prompts could ask participants to compare current experiences with earlier sessions. This design supported comparability across participants while allowing personally salient barriers and facilitators to be described [40].

Interview material was segmented into analyzable excerpts linked to participant study codes, group membership, DBF phase, theme label, and valence category. The excerpt-level approach was used because a single participant could describe several barriers and facilitators within the same interview. Segmenting these statements allowed the analysis to retain complexity while summarizing unique-participant theme endorsement at the phase level.

#### Recording and Data Transformation

Initial Chinese-language transcripts were generated from the audio recordings using a Qwen-based automated transcription tool. Participant names and direct identifiers were replaced with study codes in the analytic matrix. The original Chinese transcripts were retained as the primary textual evidence, and Chinese-to-English translations used for analysis and reporting were checked by the first author. Quotations retained in the paper were checked against the corresponding Chinese transcript and shortened, where necessary, by removing repeated fillers or unclear fragments rather than silently reconstructing them. Author-confirmed summaries from participant 6 were used for coding and participant-level counts but not as direct quotations.

### Analysis

#### Data-Analytic Strategies

Qualitative analysis followed thematic analysis and framework-oriented procedures [41-43]. A DBF-informed codebook combined phase tags (entry, adaptation, and conditional adoption) with theme labels for barriers and facilitators. Initial codes were developed from the interview guide, DBF phases, and repeated reading of excerpts. Codes were then refined to distinguish conceptually adjacent themes such as Initial Uncertainty, Physical Discomfort, Skill Adaptation, Environmental Immersion, Trust Development, and Conditional Adoption.

To improve coding transparency, the analytic matrix retained the original excerpt, English translation, DBF phase, theme label, valence, and a short coding memo. For example, comments about headset heaviness, eye discomfort, or dizziness were coded as Physical Discomfort or Motion Discomfort, depending on the specific symptom. Comments about discussing strategies, comparing scores, or sharing ways to improve were coded as Social Reinforcement. Comments that expressed willingness only if equipment comfort, floor safety, content variety, or session duration improved were coded as Conditional Adoption or Adoption With Safety Measures. A codebook excerpt and representative coding examples are provided in Multimedia Appendix 1.

Two researchers (XS and HQ) participated in coding, compared coding decisions, and resolved disagreements through discussion and consensus. Coding memos and codebook revisions were retained to support an audit trail. Thematic saturation was not treated as a statistical threshold; operationally, thematic sufficiency was judged to have been reached because later transcripts primarily elaborated existing phase-level categories, such as uncertainty, physical discomfort, immersion, social reinforcement, trust, and conditional adoption, rather than generating new dominant phase-level themes [44,45]. Theme counts represent unique participants endorsing a theme within the completed-protocol qualitative sample and are interpreted as descriptive evidence coverage rather than prevalence estimates.

#### Methodological Integrity

Methodological integrity was supported through 4 linked procedures. Adequacy was addressed by using repeated-phase prompts across the completed-protocol qualitative sample and by retaining discrepant as well as convergent accounts. Researcher perspectives were managed through the shared interview guide, explicit phase and valence fields, coding memos, comparison of coding decisions, and consensus discussion. Groundedness was supported by retaining original Chinese excerpts, linking every coded statement to a participant and session phase, and presenting representative quotations. Meaningfulness and coherence were examined through integration with questionnaire, safety, and performance evidence without forcing convergence when data sources diverged. No participant member checking was conducted, and this absence is treated as a limitation rather than implied as a completed procedure.

#### Mixed Methods Integration

Integration occurred at three levels. First, qualitative themes were quantified as unique-participant endorsements within each DBF phase. Second, theme endorsement was linked descriptively to GEQ, VRSQ, and performance summaries. Third, discrepant and illustrative cases were examined to clarify how adoption could proceed despite early uncertainty, mild symptoms, or conditional reservations. This integration strategy was intended to connect experience, performance, and interpretation without converting the study into a confirmatory quantitative comparison [46,47].

The integration priority was explanation rather than convergence alone. When interview themes, questionnaire profiles, and performance logs pointed in the same direction, they were used to strengthen interpretation. When they diverged, the qualitative account was used to clarify why a participant could improve technically while remaining cautious about comfort, safety, or future use.

#### Analytic Strategy and Quantitative Analysis

Baseline characteristics were summarized using means and SDs for continuous variables and counts and percentages for categorical variables. Standardized mean differences and absolute proportion differences were used to describe baseline imbalance. Item-level completeness, Cronbach alpha, observed score ranges, and floor and ceiling concentrations were examined for each GEQ dimension and the VRSQ. Performance was summarized at days 1, 3, and 6 for average accuracy rate and average time spent (seconds), and day 1 to day 6 change was presented with 95% CIs. Trajectory was excluded from manuscript-ready performance results because its definition and direction could not be verified. GEQ and VRSQ profiles were interpreted descriptively. No *P* values or confirmatory between-group superiority tests were reported because the study was exploratory and quasi-experimental.

#### Data Diagnostics

Data diagnostics included item completeness, observed ranges, floor and ceiling concentrations, session availability, and verification of performance-field definitions. No outlier exclusions, transformations, or imputations were used. Trajectory was excluded because its direction and operational definition could not be verified.

#### Missing Data

The completed-protocol quantitative dataset and main qualitative coded dataset each included 34 participants (VR, n=16; MR, n=18). The partial-exposure MR transcript was retained only for diagnostic reconciliation and excluded from main theme counts. One completed-protocol VR transcript was checked and added to the coding matrix using verified coding rows. Given the small sample size and the nonconfirmatory design, analyses used complete-case summaries by data source, and missingness was reported descriptively rather than modeled with imputation. A formal missing completely at random test and multiple imputation were not conducted because the study did not make confirmatory estimates.

#### Safety Monitoring

All sessions were supervised by the research team. Participants received orientation before headset use, assistance with equipment fitting, and reminders that they could pause, rest, or stop at any point. A researcher remained nearby to monitor movement safety, dizziness, fatigue, discomfort, headset pressure, anxiety, and potential fall risk. Predefined stopping criteria included participant request to stop, visible instability, worsening dizziness or nausea, marked fatigue, anxiety, headset discomfort that did not resolve after adjustment, or any researcher concern that continued movement could increase fall risk. Monitoring procedures included presession orientation, in-session observation, symptom checks during pauses or after reported discomfort, immediate rest or headset removal when needed, and postsession confirmation that symptoms had resolved or remained mild. The training area was arranged to reduce obstacles and allow researcher assistance if needed. No falls, serious adverse events, or session terminations were recorded.

### Ethical Considerations

The study received institutional approval from the Institute of Visual Informatics, Universiti Kebangsaan Malaysia, following assessment by selected institutional examiner panels on July 2, 2024 (official institutional approval reference: UKM.IVI.600-4/6/P130610). The current repeated-exposure follow-up used data collected under this approved research program. All participants received an explanation of the study purpose and procedures and provided written informed consent before participation. Study data were deidentified for analysis, participant identifiers were replaced with study codes, and no identifiable participant images are included in the study or Multimedia Appendices. No cash or performance-based financial compensation was provided. Participants received small noncash souvenirs as tokens of appreciation at predefined milestones.

## Results

### Participant Flow and Participant Characteristics

**Figure 1.**
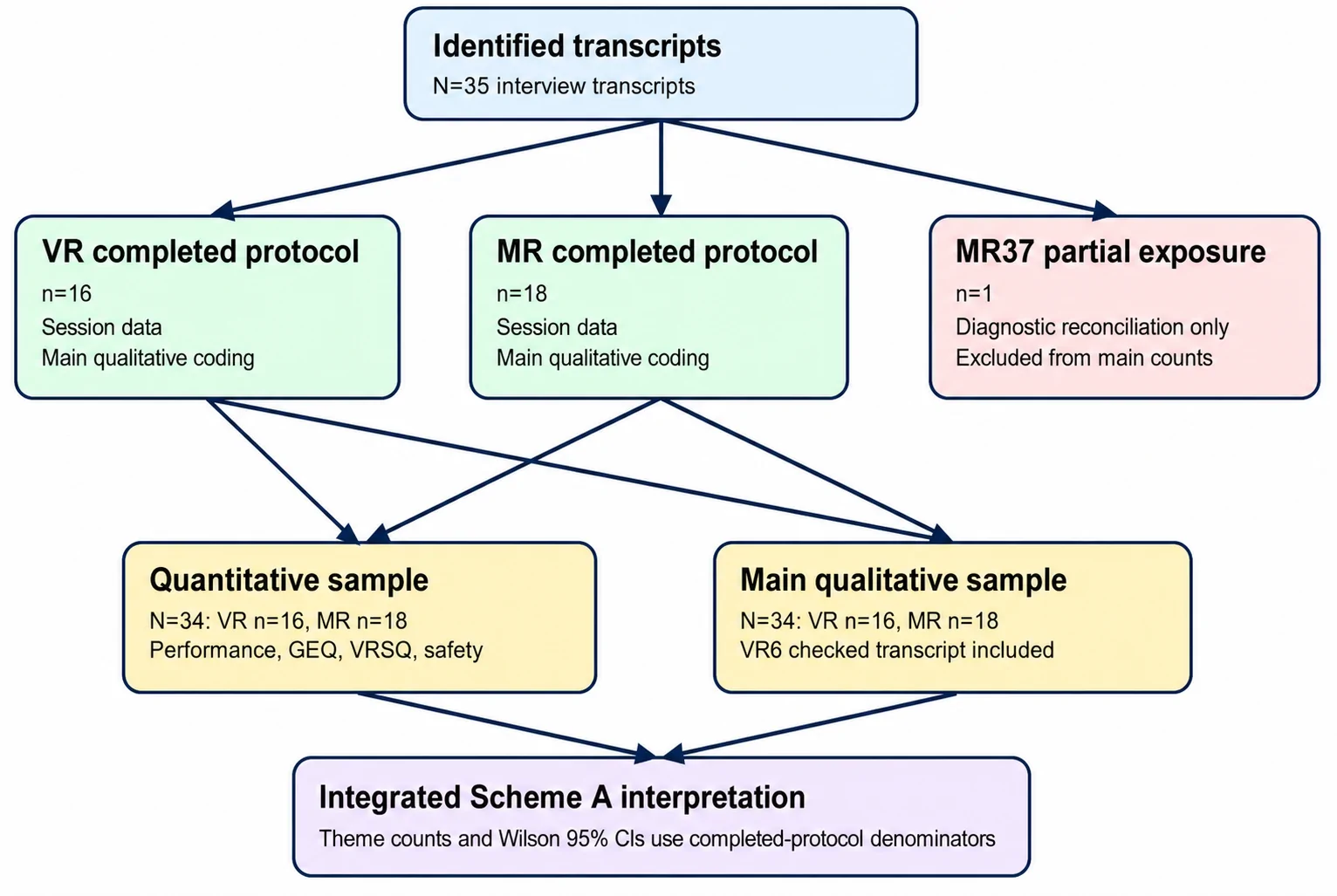
Participant flow diagram for the qualitative-dominant mixed methods follow-up study of immersive Tai Chi training among older adults in Hunan, China.

Thirty-five interview transcripts were identified. The main completed-protocol analysis included 34 participants for quantitative data (VR, n=16; MR, n=18) and 34 participants for qualitative coded evidence (VR, n=16; MR, n=18). Participant 37 was an MR partial-exposure case and was excluded from main theme counts. Participant 6 was a completed-protocol VR participant whose author-confirmed paraphrased coding was added to the qualitative coding matrix for participant-level counts but not for direct quotation. This handling creates a single denominator for the main completed-protocol interpretation. Participant disposition and analytic inclusion are summarized in Figure 1.

The flow diagram summarizes 35 identified interview transcripts, the exclusion of MR37 from the main theme counts, and the inclusion of completed-protocol VR6 coding in the main qualitative denominator. The complete quantitative sample had a mean age of 69.38 (SD 8.80; 95% CI 64.69‐74.06) years in VR and 68.00 (SD 7.17; 95% CI 64.43‐71.57) years in MR. BMI was 22.92 (SD 2.82; 95% CI 21.42‐24.43) kg/m^2^ in VR and 23.73 (SD 3.19; 95% CI 22.14‐25.32) kg/m^2^ in MR. BBS scores were high in both groups: 53.81 (SD 5.06; 95% CI 51.12‐56.51) in VR and 55.06 (SD 2.26; 95% CI 53.93‐56.18) in MR. Baseline standardized mean differences were 0.17 for age, –0.27 for BMI, and –0.32 for BBS. The groups also differed descriptively in sex distribution, smartphone use, education, and self-reported exercise intensity. These imbalances may have influenced engagement, technology confidence, safety appraisal, and trust development.

The broader recruitment pathway and participant flow are reported above. Exact calendar dates for recruitment and repeated follow-up were not available in the archived analytic files used for this revision.

### Statistics and Data Analysis

All 34 participants had complete GEQ and VRSQ item data. Reliability was good for GEQ competence (α=0.834), flow (α=0.801), and positive affect (α=0.837), acceptable for the 5-item adapted sensory and imaginative immersion dimension (α=0.711), and acceptable for the standardized VRSQ total (α=0.740). GEQ challenge was questionable (α=0.657) and was interpreted cautiously. GEQ tension and annoyance showed severe floor concentration and zero-variance items, while GEQ negative affect showed inconsistent item covariance (α=–0.442); neither was treated as a reliable composite for substantive interpretation. The standardized VRSQ total was 8.65 (SD 8.89) in VR and 5.65 (SD 7.67) in MR. These questionnaire findings provide descriptive context only and do not establish modality superiority (Table 3).

**Table 3.** Questionnaire reliability and descriptive profiles by delivery group^a^.

Scale	Items	Cronbach α	Reliability and caution	VR^b^ (n=16), mean (SD)	MR^c^ (n=18), mean (SD)
GEQ^d^ competence	5	0.834	Good	3.41 (0.69)	3.24 (0.82)
GEQ sensory and imaginative immersion (5-item adapted)	5	0.711	Acceptable for descriptive triangulation	3.69 (0.47)	2.64 (0.58)
GEQ flow	5	0.801	Good	3.83 (0.41)	2.57 (0.70)
GEQ tension and annoyance	3	0	Not interpretable: severe floor and zero variance	0.08 (0.33)	0.00 (0.00)
GEQ challenge	5	0.657	Questionable; interpret cautiously	1.30 (0.44)	1.20 (0.73)
GEQ negative affect	4	−0.442	Inconsistent item covariance; do not use as a reliable scale	0.41 (0.40)	0.33 (0.33)
GEQ positive affect	5	0.837	Good	3.74 (0.47)	3.53 (0.61)
VRSQ^e^ total (standard 0‐100 score)	9	0.740	Acceptable for descriptive triangulation	8.65 (8.89)	5.65 (7.67)

a GEQ dimensions are reported on the 0-4 analytic scale. The VRSQ total uses the standard 0-100 score. Reliability and distribution diagnostics are used to delimit interpretation, not to select favorable outcomes.

b VR: virtual reality.

c MR: mixed reality.

d GEQ:

e VRSQ: Virtual Reality Sickness Questionnaire.

### Performance Across Repeated Sessions

Both groups showed practice-related changes in system-recorded performance across supervised sessions. Average accuracy rate increased from 0.812 (SD 0.134) on day 1 to 0.895 (SD 0.088) on day 6 in VR and from 0.817 (SD 0.174) to 0.920 (SD 0.081) in MR. Day 1 to day 6 accuracy change was 0.083 (95% CI 0.024-0.142) in VR and 0.103 (95% CI 0.037-0.169) in MR. Average time spent decreased from 5.99 (SD 2.49) seconds to 5.14 (SD 0.97) seconds in VR and from 5.26 (SD 0.94) seconds to 4.75 (SD 0.60) seconds in MR. Average time spent change was −0.856 (95% CI −1.754 to 0.042) seconds in VR and −0.506 (95% CI −0.802 to −0.209) seconds in MR. These within-sample descriptive changes are compatible with increasing task familiarity and performance adjustment, but they do not establish causal learning or modality superiority (Table 4).

**Table 4.** Performance at days 1, 3, and 6 and descriptive day 1 to day 6 change.

Metric^a^	Group	Day 1, mean (SD)	Day 3, mean (SD)	Day 6, mean (SD)	Day 1 to day 6 change (95% CI)
Accuracy	VR^b^	0.812 (0.134)	0.864 (0.100)	0.895 (0.088)	0.083 (0.024 to 0.142)
Accuracy	MR^c^	0.817 (0.174)	0.858 (0.199)	0.920 (0.081)	0.103 (0.037 to 0.169)
Average time spent (in seconds)	VR	5.99 (2.49)	5.54 (1.37)	5.14 (0.97)	−0.856 (−1.754 to 0.042)
Average time spent (in seconds)	MR	5.26 (0.94)	4.99 (0.86)	4.75 (0.60)	−0.506 (−0.802 to −0.209)

a Average accuracy rate is the system-recorded proportion of logged frames in which the head and hand hotspots were within the game's tolerance window. Average time spent is reported in seconds; lower values indicate faster task performance. 95% CIs are descriptive; no *P* values or between-group superiority tests were performed.

b VR: virtual reality.

c MR: mixed reality.

### Findings

#### Phase 1: Entry, Uncertainty, and Mild Physical Discomfort

Entry-stage responses were characterized by unfamiliarity, uncertainty, and bodily adjustment. Initial Uncertainty was endorsed by 7 of 16 participants (43.8%; 95% CI 23.1%‐66.8%) in VR and 6 of 18 participants (33.3%; 95% CI 16.3%‐56.3%) in MR. Physical Discomfort was endorsed by 4 of 16 participants (25.0%; 95% CI 10.2%‐49.5%) in VR and 3 of 18 participants (16.7%; 95% CI 5.8%‐39.2%) in MR. Initial Adaptation was endorsed by 1 of 16 participants (6.2%; 95% CI 1.1%‐28.3%) in VR and 5 of 18 participants (27.8%; 95% CI 12.5%‐50.9%) in MR. These findings indicate that early resistance was not reducible to physiological discomfort alone. Participants also described unfamiliar equipment, uncertainty about whether they could operate the system, and concern about performing movements correctly.

A VR participant stated, “I was a little worried.” An MR participant described the first five minutes as “It felt very difficult.” These transcript-grounded excerpts illustrate competence and task uncertainty rather than physical intolerance alone. Other participants described novelty and curiosity, suggesting that unfamiliarity could operate as both a barrier and a facilitator depending on whether orientation and reassurance were sufficient.

This entry pattern has practical importance because it suggests that support should begin before performance feedback. Older adults who worried about operating the system or performing incorrectly needed clear orientation, slow-paced familiarization, and visible researcher support. Early adoption was also a response to whether participants felt that errors, pauses, and bodily sensations could be managed safely.

#### Phase 2: Adaptation, Immersion, and Social Reinforcement

During adaptation, participants increasingly described the training in relation to perceived improvement, environmental experience, and encouragement from others. Environmental Immersion was endorsed by 9 of 16 participants (56.2%; 95% CI 33.2%‐76.9%) in VR and 8 of 18 participants (44.4%; 95% CI 24.6%‐66.3%) in MR. Social Reinforcement was endorsed by 7 of 16 participants (43.8%; 95% CI 23.1%‐66.8%) in VR and 9 of 18 participants (50.0%; 95% CI 29.0%‐71.0%) in MR. Skill Adaptation was endorsed by 6 of 16 participants (37.5%; 95% CI 18.5%‐61.4%) in VR and 9 of 18 participants (50.0%; 95% CI 29.0%‐71.0%) in MR. Emotional Engagement was endorsed by 1 of 16 participants (6.2%; 95% CI 1.1%‐28.3%) in VR and 6 of 18 participants (33.3%; 95% CI 16.3%‐56.3%) in MR.

VR accounts often connected engagement to scenery, music, and emotional comfort. One VR participant explained, “I preferred the openness of nature, the boundless mountains...it felt as though I was immersed in nature.” MR accounts more often connected adaptation to remaining oriented to the real environment while learning the movement sequence; one participant stated, “I think I could get better the more I played.” Social reinforcement appeared in both groups. A VR participant described strategy sharing directly: “I wanted to share my method with them so that they could also achieve higher scores.” These accounts show that peer comparison and shared problem solving operated alongside immersive scenery and system feedback.

GEQ profiles supported this interpretation descriptively: VR had higher sensory and imaginative immersion (3.69; SD 0.47) and flow (3.83; SD 0.41) than MR (2.64; SD 0.58 and 2.57; SD 0.70), respectively. These questionnaire summaries are used only to contextualize qualitative interpretation, not to test modality superiority.

#### Phase 3: Trust Development and Conditional Adoption

Late-stage adoption was conditional rather than unconditional. Conditional willingness with improvements was endorsed by 6 of 16 participants (37.5%; 95% CI 18.5%‐61.4%) in VR and 1 of 18 participants (5.6%; 95% CI 1.0%‐25.8%) in MR. Conditional willingness was endorsed by 2 of 16 participants (12.5%; 95% CI 3.5%‐36.0%) in VR and 4 of 18 participants (22.2%; 95% CI 9.0%‐45.2%) in MR. Trust Development was endorsed by 0 in VR and 5 of 18 participants (27.8%; 95% CI 12.5%‐50.9%) in MR, while Trust and conditional willingness was endorsed by 5 of 16 participants (31.2%; 95% CI 14.2%‐55.6%) in VR and 3 of 18 participants (16.7%; 95% CI 5.8%‐39.2%) in MR. These patterns should be interpreted cautiously because baseline and follow-up conditions differed descriptively, but they suggest different experiential emphases.

Some participants expressed willingness to continue immersive Tai Chi if safety, headset comfort, session duration, content variety, and the physical setting were improved. An MR participant answered “I trusted it” and later described the equipment as “it felt reliable.” A VR participant described a possible place in the daily routine: “I could dance for exercise in the morning and play this in the afternoon; that would be quite good.” Another linked willingness to a practical environmental safeguard: “Just put down a thin carpet.then I would not be worried.” These accounts support conditional adoption rather than evidence of long-term unsupervised adherence.

Conditional adoption was therefore not a weak form of acceptance; it was the dominant form of late-stage evaluation in this supervised setting. Participants did not simply accept or reject the system. They specified conditions under which continued use would be reasonable, such as shorter sessions, more comfortable headset fitting, safer space arrangement, clearer guidance, or richer content (Table 5).

**Table 5.** Representative quotations supporting DBF^a^ phase interpretation in the qualitative sample^b^.

Quote ID	Group	Phase	Theme	Representative quote	Interpretive use
1_Q01	VR^c^	Phase 1	Initial Uncertainty	“I was a little worried.”	Entry-stage uncertainty stated in the transcript
7_Q01	MR^d^	Phase 1	Initial Uncertainty	“During the first five-minute round, it felt very difficult.”	Early perceived difficulty
35_Q01	VR	Phase 1‐2	Initial Adaptation	“After playing two or three times...I became more interested.”	Interest after repeated exposure
13_Q01	MR	Phase 1	Initial Uncertainty	“I had never played it before and did not know what it was like...I could not imagine it.”	Lack of a prior mental model
1_Q03	VR	Phase 2	Environmental Immersion	“I preferred the openness of nature, the boundless mountains...it felt as though I was immersed in nature.”	Environmental immersion
3_Q03	MR	Phase 2	Skill Adaptation	“Yes. I think I could get better the more I played.”	Perceived skill adaptation
4_Q05	MR	Phase 3	Trust Development	“I trusted it...it felt reliable.”	Trust appraisal after use
10_Q06	VR	Phase 3	Conditional willingness with improvements	“I could dance for exercise in the morning and play this in the afternoon; that would be quite good.”	Conditional integration into daily activity
1_Q05	VR	Phase 2	Social Reinforcement	“I wanted to share my method with them so that they could also achieve higher scores.”	Peer strategy sharing
25_Q06	VR	Phase 3	Adoption With Safety Measures	“Just put down a thin carpet...then I would not be worried.”	Practical safety condition

a DBF: Dynamic Barrier Framework.

b Quotations are shortened, transcript-grounded excerpts in the original Chinese. Repeated fillers and unclear fragments were omitted, with ellipses marking omissions; substantive wording was not added. Participant identifiers are study codes, not personal identifiers.

c VR: virtual reality.

d MR: mixed reality.

#### Mixed Methods Integration and Safety Findings

**Figure 2.**
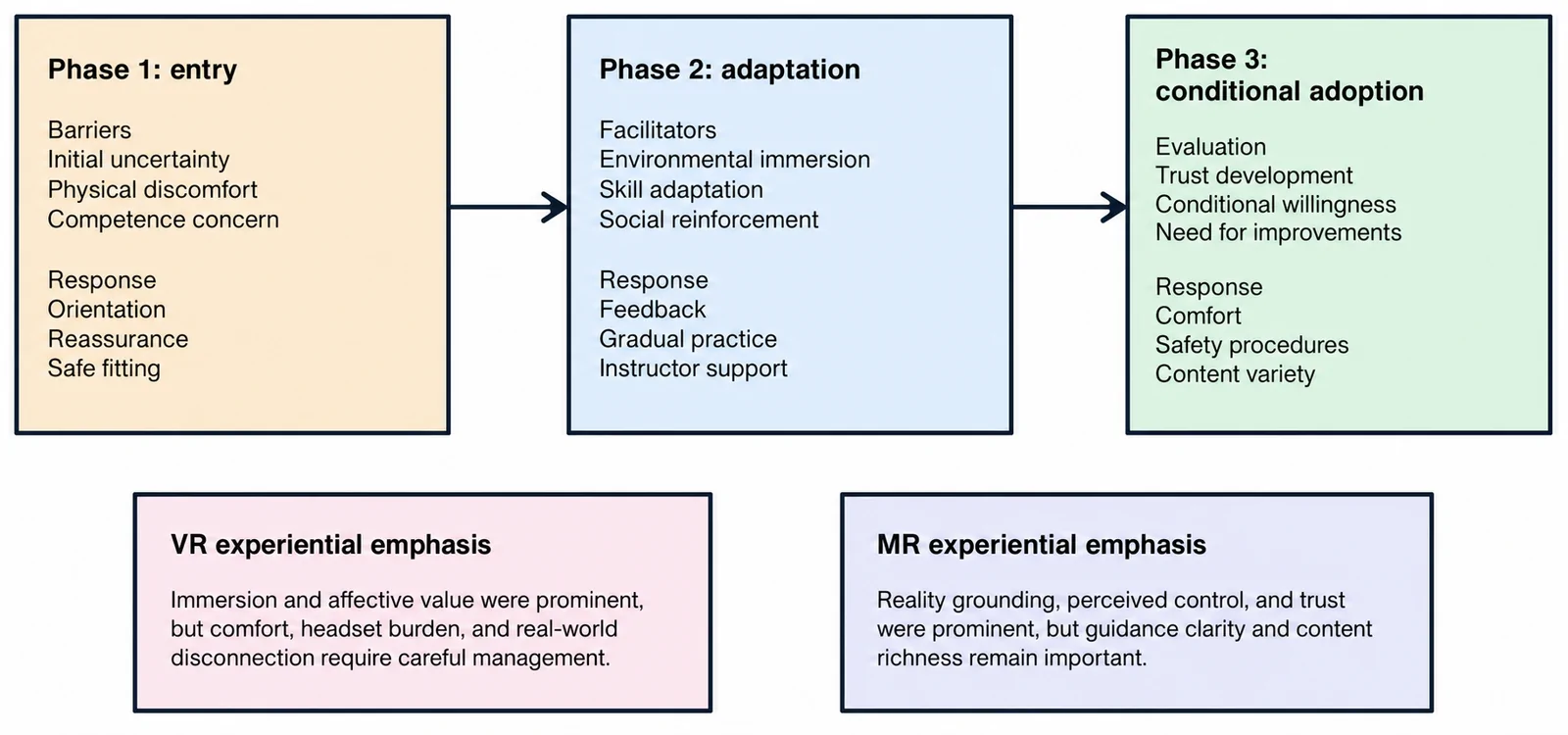
Thematic map of dynamic barriers and facilitators across Dynamic Barrier Framework phases in repeated immersive Tai Chi training.

Triangulation across interviews, questionnaires, and performance logs supported a phase-sensitive interpretation. Accuracy improved in both groups, but participants interpreted improvement through different experiential frames. VR accounts often connected engagement to environmental immersion and affective value, whereas MR accounts more often emphasized reality grounding, perceived control, and trust. The same performance pattern could be interpreted differently depending on comfort, flow, sickness symptoms, and perceived control. Figure 2 synthesizes how entry uncertainty and physical discomfort gave way to practice-supported adaptation and then to trust or conditional willingness; the arrows organize the interpretation and do not represent tested causal effects. The map is a qualitative-dominant visual synthesis.

Safety-related interview content included dizziness, fatigue, eye discomfort, headset pressure, and concern about falling. Strict safety and discomfort themes were present in 7/16 (43.8%) of VR participants and 11/18 (61.1%) of MR participants. No falls, serious adverse events, or session terminations were recorded. These mild symptoms nevertheless shaped conditional adoption because participants evaluated continued use in relation to whether the system felt safe, manageable, and worthwhile (Table 6).

**Table 6.** Main-text theme candidates by DBF^a^ phase.^b^

Phases and themes		VR^c^ (n=16)		MR^d^ (n=18)	
		Count, n (%)	95% CI	Count, n (%)	95% CI
Phase 1					
Entry					
Initial Uncertainty		7 (43.8)	23.1‐66.8	6 (33.3)	16.3‐56.3
Physical Discomfort		4 (25.0)	10.2‐49.5	3 (16.7)	5.8‐39.2
Initial Adaptation		1 (6.2)	1.1‐28.3	5 (27.8)	12.5‐50.9
Manageable Challenge		4 (25.0)	10.2‐49.5	2 (11.1)	3.1‐32.8
Initial Readiness		4 (25.0)	10.2‐49.5	0 (0)	0 (0)
Novelty Appeal		2 (12.5)	3.5‐36.0	3 (16.7)	5.8‐39.2
Phase 2					
Adaptation					
Environmental Immersion		9 (56.2)	33.2‐76.9	8 (44.4)	24.6‐66.3
Social Reinforcement		7 (43.8)	23.1‐66.8	9 (50.0)	29.0‐71.0
Skill Adaptation		6 (37.5)	18.5‐61.4	9 (50.0)	29.0‐71.0
Emotional Engagement		1 (6.2)	1.1‐28.3	6 (33.3)	16.3‐56.3
Ease of Use		3 (18.8)	6.6‐43.0	1 (5.6)	1.0‐25.8
Physical and Emotional Benefit		5 (31.2)	14.2‐55.6	0 (0)	0 (0)
Phase 3					
Conditional willingness					
Conditional willingness with improvements		6 (37.5)	18.5‐61.4	1 (5.6)	1.0‐25.8
Trust and conditional willingness		5 (31.2)	14.2‐55.6	3 (16.7)	5.8‐39.2
Conditional willingness		2 (12.5)	3.5‐36.0	4 (22.2)	9.0‐45.2
Trust development		0 (0)	0 (0)	5 (27.8)	12.5‐50.9
Willingness to continue		1 (6.2)	1.1‐28.3	2 (11.1)	3.1‐32.8
Conditional willingness with personalization		0 (0)	0 (0)	2 (11.1)	3.1‐32.8

a DBF: Dynamic Barrier Framework.

b Theme counts are unique-participant endorsements within the completed-protocol qualitative sample. They are descriptive indicators of evidence coverage and not population prevalence estimates.

c VR: virtual reality.

d MR: mixed reality.

## Discussion

### Support of Original Hypotheses and Principal Findings

No confirmatory hypotheses were specified; findings were evaluated against the stated objectives and research questions. This exploratory, qualitative-dominant longitudinal mixed methods follow-up study addressed how older adults’ barriers and facilitators evolved during repeated immersive Tai Chi training and how VR and MR delivery appeared to shape that process. Adoption unfolded from entry uncertainty and bodily adjustment, through practice- and socially supported adaptation, toward trust and conditional willingness based on comfort, perceived safety, content value, and implementation support. System logs showed higher average accuracy rate and lower average time spent over repeated supervised sessions, which is consistent with task familiarization but does not by itself demonstrate durable motor learning. Questionnaire evidence contextualized immersion, flow, and sickness without establishing modality superiority. Together, these findings answer the stated aims by showing that adoption was a changing, embodied, and socially situated evaluation rather than a single intention score or simple device preference.

### Similarity of Results: Dynamic Adoption Beyond Static Acceptance Models

The findings extend technology acceptance and continuance research by showing why repeated exposure matters for movement-based immersive systems. Existing models emphasize usefulness, ease of use, social influence, facilitating conditions, and continuance intention [18-23]. Recent older-adult studies have also documented favorable acceptance after immersive training or repeated group exposure [24,25]. This study adds a temporal and bodily explanation: usefulness and ease of use were not fixed beliefs but judgments revised as participants learned headset fitting, interpreted movement feedback, managed symptoms, compared performance, and tested whether stopping or asking for help was acceptable. The DBF therefore complements established acceptance models by specifying when and how barriers changed during supervised use. In this process, feedback-supported competence is consistent with self-efficacy theory [48], while confidence in system feedback and stopping procedures is better understood as calibrated trust than unqualified acceptance [49].

Early uncertainty had at least four forms. Procedural uncertainty concerned how to wear and operate unfamiliar equipment; performance uncertainty concerned whether movements were correct or tasks could be completed; embodied uncertainty concerned dizziness, fatigue, headset pressure, and balance; and environmental uncertainty concerned awareness of the physical room while moving. These concerns resemble recent reports that older adults need age-appropriate adaptation, clear support, suitable challenge, and socially meaningful use [24,31,32]. In this study, orientation addressed procedural uncertainty, repeated practice and feedback addressed performance uncertainty, symptom monitoring addressed embodied uncertainty, and supervision or passthrough visibility addressed environmental uncertainty. Hesitation should therefore not be equated automatically with nonacceptance; it can be a rational safety appraisal that changes when uncertainty becomes manageable.

#### Interpretation: VR and MR as Differentiated but not Superior Pathways

The VR and MR comparison should be interpreted cautiously. Recent reviews support the potential of immersive training to improve balance and physical activity outcomes in older adults [9,10], and a preliminary randomized trial reported promising findings for MR-based exercise in older women with sarcopenia [29]. Those studies establish clinical and implementation plausibility, but they do not determine whether VR or MR is superior for the present Tai Chi application. Our qualitative evidence instead identifies different experiential emphases: VR accounts foregrounded scenery, absorption, and affective value, whereas MR accounts more often foregrounded visual grounding, control, and trust. These are design hypotheses rather than causal modality effects. Alternative explanations remain plausible because baseline imbalance, prior technology experience, novelty, supervised delivery, community familiarity, and individual preference can shape immersive-system responses [11-16,50]. Preserving access to the physical room may support orientation for some users but reduce absorption, whereas full visual immersion may enhance engagement while increasing uncertainty for others [26-28,50]. Future comparisons should therefore manipulate occlusion, feedback, and supervision directly rather than treating the present group pattern as a stable property of either modality.

Social reinforcement deserves separate emphasis. Participants described discussing strategies, comparing scores, sharing methods, and wanting multiplayer or encouragement features. This is consistent with recent group and remote VR studies in which trusted facilitators, peer connection, and group delivery shaped acceptance and engagement [25,30], and with qualitative evidence that older users wanted stronger social engagement in VR exercise games [31]. For community programs, social support is therefore not an accessory to the headset. It is part of the intervention mechanism and may remain motivational after technological novelty declines.

#### Safety, Comfort, and Conditional Adoption

Safety concerns were not peripheral; they were part of the adoption process. Average VRSQ scores were low, and no falls, serious adverse events, or session terminations were recorded, but interviews still documented dizziness, fatigue, eye discomfort, headset pressure, and concern about falling. This combination is consistent with reviews describing generally feasible immersive exercise alongside persistent usability and tolerability concerns [9,10,50]. Symptom averages can therefore understate the importance of episodic discomfort for an individual’s decision to continue. Short initial sessions, stable layouts, headset-fit checks, visible stopping procedures, symptom monitoring, and instructor support serve both risk control and trust formation [9,10,50]. The absence of serious adverse events in supervised sessions is not evidence of safety for unsupervised community or home use because supervision, equipment setup, and immediate assistance were part of the conditions under which participants evaluated the system. Recent adherence research likewise notes that XR exercise evidence is dominated by short, supported programs and rarely includes long-term community follow-up [32]. Any transfer to home use should therefore be preceded by explicit risk assessment, an orientation protocol, clear stopping rules, and a plan for technical or caregiver support [32,51-53].

#### Implications for Practice

For community and home implementation, immersive Tai Chi should be delivered as a phase-sensitive service rather than a stand-alone product. Entry support should reduce procedural, performance, bodily, and environmental uncertainty through orientation, simple controls, fit checks, visible safety precautions, and permission to pause. Adaptation support should combine understandable performance feedback, progressive challenge, peer comparison, and encouragement. Conditional adoption requires lighter and easier-to-fit equipment, meaningful content variety, transparent safety procedures, and realistic access to instructor, caregiver, or technical support. These priorities align with calls for age-appropriate challenge, social engagement, and service continuity in XR exercise [31,32] and with broader evidence that older adults’ health-technology engagement depends on usability, perceived value, support, and fit with daily life [51-53]. For VR delivery, the priority is to preserve the motivational value of immersive scenery while reducing disorientation, headset burden, and uncertainty about the surrounding space [9,10,50]. For MR delivery, environmental visibility should be paired with clear virtual guidance, trustworthy feedback, and sufficient content richness; recent MR exercise evidence also supports supervised, adaptive task progression rather than unassisted exposure [29]. For both modalities, community programs should plan staff training, safe layouts, symptom checks, and gradual progression before considering independent home use [32,51-53].

#### Limitations and Generalizability

Several limitations should be considered. First, the follow-up retained the parent study allocation and was not rerandomized; the VR and MR comparison is exploratory. Second, the small community sample from Hunan, China limits transferability. Third, one MR partial-exposure case was excluded from main theme counts, and participant 6 contributed author-confirmed paraphrased coding rather than direct quotations. Fourth, baseline differences in sex distribution, smartphone use, exercise intensity, and BBS may have influenced engagement and trust. Fifth, some participants were previously known through community connections, which may have affected participation or disclosure despite standardized procedures. Sixth, automated transcription may contain recognition errors; quotations were therefore shortened to transcript-grounded excerpts and should remain subject to final source-audio checking before submission. Seventh, six supervised sessions do not demonstrate long-term adherence or unsupervised safety. Eighth, the system-defined average accuracy rate and average time spent quantify game-task performance rather than validated biomechanical technique; the backend’s numeric spatial-tolerance threshold was not preserved in the available export, and trajectory direction and definition were unverifiable. Performance findings were therefore restricted to directly reproducible descriptive fields, and trajectory was excluded. Finally, theme counts indicate evidence coverage within this qualitative sample, not population prevalence.

#### Future Directions

Future research should prospectively register larger and more balanced trials, predefine adverse-event and stopping procedures, and preserve backend metric specifications, including spatial-tolerance thresholds, before data collection. Repeated-measures designs should distinguish short-term task familiarization from retained motor learning and should test whether phase-tailored orientation, feedback, social support, and symptom management improve adherence. VR and MR comparisons should hold exercise content constant while manipulating environmental visibility and feedback, and they should include longer community or home follow-up. This agenda is consistent with current evidence calling for multidimensional adherence measurement, long-term tracking, and direct evaluation of age-sensitive XR service design [9,29,32].

### Conclusion

This exploratory, qualitative-dominant longitudinal mixed methods follow-up study is innovative in combining repeated qualitative accounts with symptom, safety, and performance evidence while holding the Tai Chi content constant across VR and MR delivery. Unlike single-session acceptance studies, broad XR comparisons, or endpoint-only evaluations, it shows that older adults repeatedly evaluate whether an immersive system is understandable, physically manageable, socially supported, and worth continuing. The DBF-informed account contributes a phase-sensitive explanation of how entry barriers may soften, persist, or change meaning and how VR and MR affordances can shape experience without establishing device superiority. The real-world implication is that immersive exercise should be implemented as an age-friendly service rather than a stand-alone headset: programs need gradual orientation, monitored exposure, comfort and safety safeguards, understandable feedback, meaningful challenge, social support, and modality-sensitive design [9,10,29,31,32,51-53]. Longer community or home follow-up remains necessary before inferring sustained adherence or unsupervised safety.

## Supplementary material

10.2196/96262 Multimedia Appendix 1 Supplementary figures and tables for the qualitative-dominant mixed methods follow-up study of immersive tai chi training among older adults.

## Abbreviations

AR: augmented reality
BBS: Berg Balance Scale
DBF: Dynamic Barrier Framework
GEQ: Game Experience Questionnaire
MR: mixed reality
TAM: technology acceptance model
UTAUT: unified theory of acceptance and use of technology
VR: virtual reality
VRSQ: Virtual Reality Sickness Questionnaire
XR: extended reality
