# Supplementary material for: Dynamic Adoption of Immersive Tai Chi Training Among Older Adults: Exploratory Qualitative-Dominant Longitudinal Mixed Methods Follow-Up Study of Virtual and Mixed Reality Delivery
Source: JMIR Serious Games. 2026 Sep 15;14:e96262. doi: 10.2196/96262 (PMC13576671; doi:10.2196/96262)
Supplement: Supplementary file 1 [file games-v14-e96262-s001.docx]

# Multimedia Appendix 1

Supplementary figures and tables for the qualitative-dominant longitudinal mixed-methods study of immersive Tai Chi training among older adults.

Unless otherwise noted, the completed-protocol quantitative/session sample and main qualitative coded sample each use VR n=16 and MR n=18. Participant 37 was retained only for diagnostic reconciliation and excluded from main theme counts. Participant 6 contributed author-confirmed paraphrased coding to participant-level theme counts but was not eligible for use as a verbatim quotation.

# Figure S1. Overview of VR/MR delivery modes and study procedure.

Note. Both groups used the same Tai Chi exergame and session structure; delivery mode was the intended difference.

# Figure S2. Subjective experience and sickness profile by group (GEQ and VRSQ).

Note. Points show group means with 95% confidence intervals. GEQ dimensions use 0-4 scoring. VRSQ total uses the standard 0-100 score; its panel is displayed from 0 to 30 for readability. GEQ=Game Experience Questionnaire; VRSQ=Virtual Reality Sickness Questionnaire.

# Table S1. Baseline/context descriptive variables.

| Variable | Level | Overall (N=34) | VR (n=16) | MR (n=18) |
| --- | --- | --- | --- | --- |
| Age, years | — | 68.65 (7.89) | 69.38 (8.80) | 68.00 (7.17) |
| Gender, n (%) | Male | 10 (29.4) | 7 (43.8) | 3 (16.7) |
|  | Female | 24 (70.6) | 9 (56.2) | 15 (83.3) |
| BMI, kg/m² | — | 23.35 (3.01) | 22.92 (2.82) | 23.73 (3.19) |
| Education, n (%) | Primary school or below | 4 (11.8) | 2 (12.5) | 2 (11.1) |
|  | Middle school | 13 (38.2) | 4 (25.0) | 9 (50.0) |
|  | High school/technical secondary | 13 (38.2) | 7 (43.8) | 6 (33.3) |
|  | Junior college or above | 4 (11.8) | 3 (18.8) | 1 (5.6) |
| Daily smartphone use, n (%) | <1 hour | 4 (11.8) | 2 (12.5) | 2 (11.1) |
|  | 1–3 hours | 9 (26.5) | 3 (18.8) | 6 (33.3) |
|  | 3–5 hours | 12 (35.3) | 9 (56.2) | 3 (16.7) |
|  | ≥5 hours | 9 (26.5) | 2 (12.5) | 7 (38.9) |
| BBS | — | 54.47 (3.83) | 53.81 (5.06) | 55.06 (2.26) |
| Exercise intensity, n (%) | Low | 6 (17.6) | 5 (31.2) | 1 (5.6) |
|  | Light | 9 (26.5) | 3 (18.8) | 6 (33.3) |
|  | Vigorous | 19 (55.9) | 8 (50.0) | 11 (61.1) |
| Health status, n (%) | No diagnosed disease | 14 (41.2) | 6 (37.5) | 8 (44.4) |
|  | Cardiopulmonary disease | 20 (58.8) | 10 (62.5) | 10 (55.6) |

Note. Values are mean (SD) or n (%). Percentages use group-specific denominators.

# Table S2. GEQ/VRSQ descriptive profile and item-level reliability diagnostics.

| Dimension | Interpretation cue | VR mean (SD) | MR mean (SD) | VR median [IQR] | MR median [IQR] |
| --- | --- | --- | --- | --- | --- |
| GEQ competence | Perceived movement and task competence | 3.41 (SD 0.69) | 3.24 (SD 0.82) | 3.70 [IQR 3.00 to 4.00] | 3.60 [IQR 2.65 to 3.80] |
| GEQ sensory and imaginative immersion (5-item adapted) | Immersive environmental experience | 3.69 (SD 0.47) | 2.64 (SD 0.58) | 3.80 [IQR 3.60 to 4.00] | 2.60 [IQR 2.45 to 2.95] |
| GEQ flow | Absorption during training | 3.83 (SD 0.41) | 2.57 (SD 0.70) | 4.00 [IQR 3.95 to 4.00] | 2.70 [IQR 1.95 to 3.00] |
| GEQ tension/annoyance | Irritation or tension during play | 0.08 (SD 0.33) | 0.00 (SD 0.00) | 0.00 [IQR 0.00 to 0.00] | 0.00 [IQR 0.00 to 0.00] |
| GEQ challenge | Perceived task challenge | 1.30 (SD 0.44) | 1.20 (SD 0.73) | 1.30 [IQR 1.00 to 1.65] | 1.30 [IQR 0.80 to 1.55] |
| GEQ negative affect | Negative affective response | 0.41 (SD 0.40) | 0.33 (SD 0.33) | 0.50 [IQR 0.00 to 0.75] | 0.25 [IQR 0.00 to 0.50] |
| GEQ positive affect | Positive affective response | 3.74 (SD 0.47) | 3.53 (SD 0.61) | 4.00 [IQR 3.70 to 4.00] | 3.80 [IQR 3.40 to 4.00] |
| VRSQ total (standard 0-100 score) | Cybersickness symptom burden | 8.65 (SD 8.89) | 5.65 (SD 7.67) | 7.92 [IQR 0.00 to 15.80] | 1.67 [IQR 0.00 to 8.12] |

Supplementary Table S2b. Item-level completeness and internal-consistency diagnostics.

| Scale | Complete n | Items | Cronbach alpha | Reliability/caution |
| --- | --- | --- | --- | --- |
| GEQ competence | 34 | 5 | 0.834 | Good |
| GEQ sensory and imaginative immersion (5-item adapted) | 34 | 5 | 0.711 | Acceptable for descriptive triangulation |
| GEQ flow | 34 | 5 | 0.801 | Good |
| GEQ tension/annoyance | 34 | 3 | 0.000 | Not interpretable: severe floor/zero variance |
| GEQ challenge | 34 | 5 | 0.657 | Questionable; interpret cautiously |
| GEQ negative affect | 34 | 4 | -0.442 | Inconsistent item covariance; do not treat as reliable |
| GEQ positive affect | 34 | 5 | 0.837 | Good |
| VRSQ total (standard 0-100 score) | 34 | 9 | 0.740 | Acceptable for descriptive triangulation |

Note. No missing item values were observed in the completed-protocol sample. Reliability estimates are diagnostic because these measures were used for descriptive triangulation rather than confirmatory psychometric inference.

# Table S3. DBF theme endorsement matrix by phase with 95% CIs.

| Phase | Theme | Total unique participants | VR | MR |
| --- | --- | --- | --- | --- |
| Phase 1: Entry | Initial Uncertainty | 13/34 (38.2%; 95% CI 23.9%-55.0%) | 7/16 (43.8%; 95% CI 23.1%-66.8%) | 6/18 (33.3%; 95% CI 16.3%-56.3%) |
| Phase 1: Entry | Physical Discomfort | 7/34 (20.6%; 95% CI 10.3%-36.8%) | 4/16 (25.0%; 95% CI 10.2%-49.5%) | 3/18 (16.7%; 95% CI 5.8%-39.2%) |
| Phase 1: Entry | Initial Adaptation | 6/34 (17.6%; 95% CI 8.3%-33.5%) | 1/16 (6.2%; 95% CI 1.1%-28.3%) | 5/18 (27.8%; 95% CI 12.5%-50.9%) |
| Phase 1: Entry | Manageable Challenge | 6/34 (17.6%; 95% CI 8.3%-33.5%) | 4/16 (25.0%; 95% CI 10.2%-49.5%) | 2/18 (11.1%; 95% CI 3.1%-32.8%) |
| Phase 1: Entry | Initial Readiness | 4/34 (11.8%; 95% CI 4.7%-26.6%) | 4/16 (25.0%; 95% CI 10.2%-49.5%) | 0 |
| Phase 1: Entry | Novelty Appeal | 5/34 (14.7%; 95% CI 6.4%-30.1%) | 2/16 (12.5%; 95% CI 3.5%-36.0%) | 3/18 (16.7%; 95% CI 5.8%-39.2%) |
| Phase 2: Adaptation | Environmental Immersion | 17/34 (50.0%; 95% CI 34.1%-65.9%) | 9/16 (56.2%; 95% CI 33.2%-76.9%) | 8/18 (44.4%; 95% CI 24.6%-66.3%) |
| Phase 2: Adaptation | Social Reinforcement | 16/34 (47.1%; 95% CI 31.5%-63.3%) | 7/16 (43.8%; 95% CI 23.1%-66.8%) | 9/18 (50.0%; 95% CI 29.0%-71.0%) |
| Phase 2: Adaptation | Skill Adaptation | 15/34 (44.1%; 95% CI 28.9%-60.5%) | 6/16 (37.5%; 95% CI 18.5%-61.4%) | 9/18 (50.0%; 95% CI 29.0%-71.0%) |
| Phase 2: Adaptation | Emotional Engagement | 7/34 (20.6%; 95% CI 10.3%-36.8%) | 1/16 (6.2%; 95% CI 1.1%-28.3%) | 6/18 (33.3%; 95% CI 16.3%-56.3%) |
| Phase 2: Adaptation | Ease of Use | 4/34 (11.8%; 95% CI 4.7%-26.6%) | 3/16 (18.8%; 95% CI 6.6%-43.0%) | 1/18 (5.6%; 95% CI 1.0%-25.8%) |
| Phase 2: Adaptation | Physical & Emotional Benefit | 5/34 (14.7%; 95% CI 6.4%-30.1%) | 5/16 (31.2%; 95% CI 14.2%-55.6%) | 0 |
| Phase 3: Conditional willingness | Conditional willingness with improvements | 7/34 (20.6%; 95% CI 10.3%-36.8%) | 6/16 (37.5%; 95% CI 18.5%-61.4%) | 1/18 (5.6%; 95% CI 1.0%-25.8%) |
| Phase 3: Conditional willingness | Trust and conditional willingness | 8/34 (23.5%; 95% CI 12.4%-40.0%) | 5/16 (31.2%; 95% CI 14.2%-55.6%) | 3/18 (16.7%; 95% CI 5.8%-39.2%) |
| Phase 3: Conditional willingness | Conditional willingness | 6/34 (17.6%; 95% CI 8.3%-33.5%) | 2/16 (12.5%; 95% CI 3.5%-36.0%) | 4/18 (22.2%; 95% CI 9.0%-45.2%) |
| Phase 3: Conditional willingness | Trust Development | 5/34 (14.7%; 95% CI 6.4%-30.1%) | 0 | 5/18 (27.8%; 95% CI 12.5%-50.9%) |
| Phase 3: Conditional willingness | Willingness to continue | 3/34 (8.8%; 95% CI 3.0%-23.0%) | 1/16 (6.2%; 95% CI 1.1%-28.3%) | 2/18 (11.1%; 95% CI 3.1%-32.8%) |
| Phase 3: Conditional willingness | Conditional willingness with personalization | 2/34 (5.9%; 95% CI 1.6%-19.1%) | 0 | 2/18 (11.1%; 95% CI 3.1%-32.8%) |

Note. Counts are unique participants endorsing a consolidated theme at least once within the relevant DBF phase. Participant 6 author-confirmed paraphrases contribute to participant-level counts but not quotation tables. Percentages and Wilson 95% CIs use VR n=16 and MR n=18 denominators.

# Table S4. Performance profile and Day 1 to Day 6 descriptive change.

| Metric | Group | Day 1, mean (SD) | Day 3, mean (SD) | Day 6, mean (SD) | Day 1 to Day 6 change (95% CI) |
| --- | --- | --- | --- | --- | --- |
| Accuracy | VR | 0.812 (0.134) | 0.864 (0.100) | 0.895 (0.088) | 0.083 (0.024 to 0.142) |
| Accuracy | MR | 0.817 (0.174) | 0.858 (0.199) | 0.920 (0.081) | 0.103 (0.037 to 0.169) |
| Average Time Spent, s | VR | 5.99 (2.49) | 5.54 (1.37) | 5.14 (0.97) | -0.856 (-1.754 to 0.042) |
| Average Time Spent, s | MR | 5.26 (0.94) | 4.99 (0.86) | 4.75 (0.60) | -0.506 (-0.802 to -0.209) |

Note. Accuracy and Average Time Spent are system-defined session-level task metrics. Positive accuracy change indicates improved accuracy; negative time change indicates faster completion. Values are descriptive and should not be interpreted as confirmatory between-group effects. Trajectory was excluded because its direction and operational definition could not be verified in the available export.

# Table S5. Representative transcript-grounded quotation examples and safety/discomfort verification tables.

| Quote ID | Group | Phase | Theme | Representative quote | Interpretive use |
| --- | --- | --- | --- | --- | --- |
| 1_Q01 | VR | Phase 1 | Initial Uncertainty | I was a little worried. | Entry-stage uncertainty stated in the transcript |
| 7_Q01 | MR | Phase 1 | Initial Uncertainty | During the first five-minute round, it felt very difficult. | Early perceived difficulty |
| 35_Q01 | VR | Phase 1-2 | Initial Adaptation | After playing two or three times...I became more interested. | Interest after repeated exposure |
| 13_Q01 | MR | Phase 1 | Initial Uncertainty | I had never played it before and did not know what it was like...I could not imagine it. | Lack of a prior mental model |
| 1_Q03 | VR | Phase 2 | Environmental Immersion | I preferred the openness of nature, the boundless mountains...it felt as though I was immersed in nature. | Environmental immersion |
| 3_Q03 | MR | Phase 2 | Skill Adaptation | Yes. I think I could get better the more I played. | Perceived skill adaptation |
| 4_Q05 | MR | Phase 3 | Trust Development | I trusted it...it felt reliable. | Trust appraisal after use |
| 10_Q06 | VR | Phase 3 | Conditional willingness with improvements | I could dance for exercise in the morning and play this in the afternoon; that would be quite good. | Conditional integration into daily activity |
| 1_Q05 | VR | Phase 2 | Social Reinforcement | I wanted to share my method with them so that they could also achieve higher scores. | Peer strategy sharing |
| 25_Q06 | VR | Phase 3 | Adoption With Safety Measures | Just put down a thin carpet...then I would not be worried. | Practical safety condition |

Note. English translations were checked by the first author against the retained Chinese transcripts and shortened only by removing filler or unclear fragments. Participant identifiers are study codes. Participant 6 paraphrases are documented separately and are not presented as quotations.

Table S5b. Strict safety/discomfort participant summary.

| Group | Participants with strict safety/discomfort mention |
| --- | --- |
| VR | 7/16 (43.8%) |
| MR | 11/18 (61.1%) |

Table S5c. Strict safety/discomfort transcript-evidence verification list.

| Quote ID | Group | DBF phase | Theme | Original Chinese excerpt | English translation | Code | Verification memo |
| --- | --- | --- | --- | --- | --- | --- | --- |
| 1_Q02 | VR | Phase 1 | Physical Discomfort | 头盔不晕，但戴久了觉得有点重、会不舒服。 | The headset did not make me dizzy, but after wearing it for a long time it felt heavy and uncomfortable. | 头盔偏重 | 初期物理负担影响舒适度 |
| 10_Q02 | VR | Phase 1 | Physical Discomfort | 第一次戴眼睛有点糊、头盔有点重，但很快就适应了。 | The first time, the display looked a little blurry and the headset felt somewhat heavy, but I adapted quickly. | 初期模糊/重量负担 | 初期轻度不适，适应后影响降低 |
| 21_Q02 | VR | Phase 1 | Health Risk Concern | 会担心设备对身体有没有影响（比如辐射/副作用）。 | I worried whether the device might affect the body, for example through radiation or side effects. | 健康风险担忧 | 初期风险感知（对身体影响的担心） |
| 25_Q06 | VR | Phase 3 | Adoption With Safety Measures | 建议地面加地毯/泡沫防摔；内容可更丰富；长期适合间接性一周两三次，愿意作为日常锻炼一部分。 | I suggested mats or foam for fall prevention and richer content; long term, two or three intermittent sessions per week would be suitable as part of daily exercise. | 地面安全改进+间歇性采纳 | 采纳意愿明确，但强调“安全防护+间歇频率+内容优化” |
| 27_Q02 | VR | Phase 1 | Physical Discomfort | 戴久了有一点点不舒服（时间长会有感觉）。 | Wearing it for a long time felt slightly uncomfortable. | 佩戴时长不适 | 初期存在轻度物理负担（随时长上升） |
| 35_Q02 | VR | Phase 1 | Physical Discomfort | 第一次戴头盔有一点压迫感；更希望是“眼镜式”会更舒服。 | The headset felt a little pressing the first time; glasses-style equipment would be more comfortable. | 压迫感/偏好眼镜式 | 初期物理不适与形态偏好影响舒适度 |
| 3_Q02 | MR | Phase 1 | Physical Adjustment Challenge | 戴头盔时眼睛有点不舒服。 | My eyes felt a little uncomfortable when wearing the headset. | 设备物理不适 | 物理层面的轻度不适 |
| 4_Q02 | MR | Phase 1 | Physical Fatigue | 玩完之后脚有点痛，有些疲劳。 | After playing, my feet hurt a little and I felt somewhat tired. | 身体疲劳/不适 | 生理层面的适应负担 |
| 7_Q02 | MR | Phase 1 | Physical Discomfort | 开始戴头盔觉得有点重，后来适应了。 | At first the headset felt a little heavy, but I adapted later. | 设备重量感知 | 初期物理负担但可适应 |
| 9_Q02 | MR | Phase 1 | Physical Discomfort | 头盔稍微有点重，但还能接受。 | The headset was slightly heavy, but acceptable. | 设备重量感知 | 存在轻度物理负担但不构成阻碍 |
| 11_Q01 | MR | Phase 1 | Physical Discomfort | 第一次戴头盔有点重，额头有点压迫感。 | The headset felt a little heavy the first time and pressed slightly on my forehead. | 设备重量感知 | 初期存在轻度物理不适 |
| 13_Q02 | MR | Phase 1 | Motion Discomfort | 取下头盔后有点头晕，眼睛有点模糊。 | After taking off the headset, I felt a little dizzy and my eyes were a little blurry. | 眩晕不适 | 存在VR生理适应问题 |
| 14_Q06 | MR | Phase 3 | Safety & Health Concern | 担心转得快会晕；长期用可能影响视力，时间不能太长。 | I worried that turning quickly could cause dizziness; long-term use might affect eyesight, so duration should not be too long. | 眩晕/视力担忧 | 长期采纳受到健康风险感知约束，需要控制强度与时长 |
| 18_Q01 | MR | Phase 1 | Motion Discomfort | 第一次戴头盔有点晕，从来没接触过，心里有点怕。 | The first time wearing the headset, I felt a little dizzy; I had never used it before and felt somewhat afraid. | 初次眩晕/陌生恐惧 | 进入阶段主要障碍是眩晕与陌生感 |
| 22_Q01 | MR | Phase 1 | Health Risk Concern | 没玩之前担心电子产品有辐射、对身体不好。 | Before using it, I worried that electronic products might have radiation and be bad for the body. | 辐射担忧 | 进入阶段的风险感知（健康风险/辐射） |
| 24_Q06 | MR | Phase 3 | Physical Discomfort | 头盔重、绑带复杂，最好更轻巧、更好戴，自己也更容易独立佩戴。 | The headset was heavy and the straps were complicated; it should be lighter and easier to put on independently. | 重量与佩戴复杂 | 长期采纳受物理负担与穿戴复杂度影响 |
| 28_Q01 | MR | Phase 1 | Motion & Physical Discomfort | 第一次戴头盔感觉有点昏，还觉得比较重。 | The first time wearing the headset, I felt a little dizzy and also thought it was quite heavy. | 初次眩晕/重量负担 | 进入阶段出现眩晕与物理负担感 |

Table S5d. Participant 6 author-confirmed paraphrased coding evidence.

| Evidence ID | Group | Phase | Theme | Author-confirmed paraphrased meaning | Use restriction |
| --- | --- | --- | --- | --- | --- |
| 6_Q01_manual | VR | Phase 1 | No Prior Expectation | No particular expectation before use. | Coding/counts only; not a verbatim quotation. |
| 6_Q02_manual | VR | Phase 1 | Self-efficacy Confidence | No concern about being unable to complete the task. | Coding/counts only; not a verbatim quotation. |
| 6_Q03_manual | VR | Phase 1 | Motion Discomfort Concern | Concern about dizziness during use. | Coding/counts only; not a verbatim quotation. |
| 6_Q04_manual | VR | Phase 2 | Exercise-Oriented Entry | Perceived the training as having exercise value. | Coding/counts only; not a verbatim quotation. |
| 6_Q05_manual | VR | Phase 3 | Trust and conditional willingness | Trusted the device, considered it suitable for older adults, and was willing to continue use. | Coding/counts only; not a verbatim quotation. |
| 6_Q06_manual | VR | Phase 2 | Initial Adaptation | Reported no obvious headset disadvantages and felt able to adapt to its weight. | Coding/counts only; not a verbatim quotation. |

# Table S6. Sample DBF codebook excerpt illustrating phase tags, theme labels, definitions, coding rules, and example excerpts.

| DBF phase | Theme label | Definition | Coding rule | Example excerpt | Valence |
| --- | --- | --- | --- | --- | --- |
| Phase 1: Entry | Initial Uncertainty | Early concern about ability, task difficulty, or unfamiliar equipment. | Code worry, hesitation, unfamiliarity, or concern about doing the task incorrectly. | I was a little worried. | Barrier |
| Phase 1: Entry | Physical Discomfort | Headset- or movement-related bodily discomfort during early use. | Code headset heaviness, pressure, eye discomfort, dizziness, fatigue, or similar discomfort. | At first the headset felt a little heavy, but I adapted later. | Barrier |
| Phase 2: Adaptation | Environmental Immersion | Engagement or affective value linked to scenery, music, or perceived presence. | Code being drawn into the scene or enjoying landscape, music, or presence. | It felt as though I was immersed in nature. | Facilitator |
| Phase 2: Adaptation | Social Reinforcement | Motivation from peers, comparison, discussion, encouragement, or shared strategies. | Code comparing scores, discussing improvement, sharing methods, or peer encouragement. | I wanted to share my method with them. | Facilitator |
| Phase 3: Conditional willingness | Trust Development | Later confidence that the equipment or procedure is reliable and manageable. | Code explicit reliability, reduced worry, or confidence after repeated exposure. | I trusted it...it felt reliable. | Facilitator |
| Phase 3: Conditional willingness | Conditional willingness with improvements | Future willingness dependent on safety, comfort, content, duration, or environmental improvements. | Code willingness conditional on safeguards or practical improvements. | Just put down a thin carpet...then I would not be worried. | Facilitator |

Note. DBF=Dynamic Barrier Framework. Example excerpts are checked English translations of retained Chinese transcript segments. Participant 6 paraphrases are excluded from this quotation-based codebook excerpt. The table is a transparency aid and does not exhaust the full coding matrix.
